# Epigenetic memories induced by hypoxia in AKI-to-CKD transition

**DOI:** 10.1007/s10157-025-02745-1

**Published:** 2025-08-20

**Authors:** Giyoung Kwoun, Masaomi Nangaku, Imari Mimura

**Affiliations:** Division of Nephrology and Endocrinology, The University of Tokyo Hospital, 7-3-1 Hongo, Bunkyo-ku, Tokyo 113-8655 Japan

**Keywords:** Histone, Hypoxic memory, AKI-to-CKD transition

## Abstract

Chronic kidney disease (CKD) is a global health burden associated with increasing mortality rates. Aging populations and declining fertility rates exacerbate this issue, particularly in countries such as Japan. Acute kidney injury (AKI) was previously considered temporary and reversible condition. However, in recent years, multiple studies on kidney diseases have shown that AKI survivors are at an increased risk of developing CKD. During the AKI-to-CKD transition, a subset of AKI-induced epigenetic alterations persists in cells, potentially driving the progression of tubulointerstitial fibrosis. Therefore, targeting epigenetic mechanisms may represent a promising therapeutic approach for preventing AKI-to-CKD transition. Among the epigenetic mechnisms involved, “hypoxic memory” plays a crucial role in this transition by inducing persistent epigenetic changes. Hypoxic memory induces DNA methylation, histone modification, changes in chromatin conformation, and long non-codingRNA (lncRNA) expression. Herein, we review the latest evidence on epigenetic memory in the AKI-to-CKD transition, identifying that the detailed mechanisms of epigenetic memory and temporal specificity are crucial for developing effective treatments.

## Introduction

Chronic kidney disease (CKD) is a progressive condition that affects over 800 million individuals (over 10% of the global population). CKD has emerged as a leading cause of mortality worldwide and is one of the few non-communicable diseases that has witnessed an increase in associated deaths within the past two decades [1]. It is known that the “memory” of AKI leads to progression to CKD; epidemiological studies show that AKI survivors are at high risk of developing CKD [2]. Also, several recent studies have demonstrated a link between AKI, cardiovascular events, and mortality, independent of CKD [3, 4]. Given that both CKD and AKI are associated with a high mortality rate, prevention of the AKI-to-CKD transition is an important issue [5]. Epigenetic memory can be defined as the ability of cells to retain and transmit altered gene expression patterns induced by prior stimuli, mediated by epigenetic marks such as DNA methylation, noncoding RNAs, chromatin conformational changes, and histone modifications [6, 7]. This memory is established during development or in response to environmental factors [7], and these changes in gene expression previously thought to be restorative, however, they have been found to persist even after the initial stimulus is gone. For example, during the course of AKI-to-CKD transition, hypoxia-induced epigenetic changes are recorded in the cell and have a long-term effect, known as “hypoxic memory” [8, 9]. Epigenetic memory is thought to help cells respond more efficiently to recurring stimuli, and give advantageous effect on cell survival. Herein, we focus on the epigenetic memories in the AKI-to-CKD transition and the persistent epigenetic alterations triggered by AKI that contribute to the subsequent development of CKD.

## Overview of the epigenetic mechanisms

Epigenetic features regulate gene expression by changing the chromatin structure or the accessibility of genetic loci via the transcriptional machinery.

### Histone modification

Chromatin is a complex structure that is formed when DNA is packaged within cells. The basic unit of chromatin is the nucleosome, which consists of 147 bp of DNA wrapped around an octamer of four core histones (H2A, H2B, H3, and H4). These histones, particularly their N-terminal ‘tails’,’ can undergo various modifications, including acetylation, methylation, phosphorylation, and ubiquitination. These modifications play a crucial role in regulating gene expression and other cellular processes [10] through various enzymes that introduce (writers), recognize (readers), or remove (erasers) them [11].

Histone acetylation involves the addition of a negatively charged acetyl group to the lysine of core histones by histone acetyltransferases (HATs), and is one of the most extensively studied forms of histone modification [11]. Acetylation of lysine residues disrupts electrostatic interactions between histones and DNA, leading to a relaxed chromatin conformation. This relaxed state allows transcription factors to gain increased access to their target genes, thereby promoting gene expression [12]. Acetylation is reversible and catalyzed by HATs, whereas deacetylation is catalyzed by histone deacetylases (HDACs). The opposing activities of HATs and HDACs tightly regulate gene expression and are correlated with gene activation and repression/silencing [13]. HDAC inhibitors (HDACis) are used as anticancer [14, 15] and antifibrogenic agents [16]. These small-molecule epigenetic modulators, categorized based on their chemical structures (e.g., short-chain fatty acids, amino-benzamides, cyclic peptides, and hydroxamic acids), offer a diverse approach for treating both hematological and solid tumors as well as non-neoplastic conditions [17]. Among these, hydroxamic acid-based HDACis, including vorinostat, romidepsin, belinostat, and panobinostat, were the first to gain approval from the US Food and Drug Administration for cancer treatment [18]. Vorinostat was the first HDACi approved by the FDA in 2006 against advanced primary cutaneous T-cell lymphoma (CTCL) and has been shown to inhibit tumor growth in different cancers [19]. Thus, it is important to elucidate the mechanism of cancer progression in terms of histone modifications, as this will aid in the development of novel anticancer drugs.

### DNA methylation

In mammals, DNA methylation is a process in which a methyl group is added to cytosine residues in the DNA, influencing gene expression. It plays a critical role in regulating various biological processes, including gene expression, X chromosome inactivation, and genomic imprinting. DNA methylation has also been linked to complex human diseases, including cancer. This modification occurs at cytosine bases within the dinucleotide sequence 5′CpG3′. CpG refers to cytosine and guanine separated by a phosphate, which links the two nucleotides in DNA [20].

This process is catalyzed by DNA methyltransferases (DNMTs) [21, 22]. DNA methylation and demethylation at specific CpG sites are essential for normal kidney development. However, abnormal methylation patterns such as hypermethylation or hypomethylation can contribute to kidney disease [23]. For example, specific changes in DNA methylation have been detected in patients with CKD, and diabetic nephropathy [24]. Since this analysis was conducted on whole blood, further analysis on specific organ tissue may be needed. The reversibility of these epigenetic modifications in human cancers makes targeting DNMTs an attractive strategy for cancer treatment. Traditional DNA hypomethylating agents (HMAs), such as decitabine (DAC) and azacytidine (AZA), have been clinically used for the treatment of hematologic malignancies [25]. HMAs reverse abnormal gene silencing in myeloid neoplasms by inhibiting enzymes that demethylate aberrantly methylated promoter regions [26]. Azacitidine, the first FDA-approved drug of this type for myelodysplastic syndrome in 2004, is now widely used for other related myeloid neoplasms. Azacitidine inhibits DNMT, causing demethylation; in DNA, it irreversibly binds to DNMT, leading to the loss of its activity [27], which results in the demethylation of genomic DNA.

### Mutual interactions between DNA methylation and histone modifications

It has become apparent that DNA methylation and histone modifications influence each other. Histone methylation contributes to guiding DNA methylation, while DNA methylation, in turn, can provide a post-replication template for specific histone modifications. The presence or absence of DNA methylation directs how DNA is assembled into chromatin. Unmethylated DNA tends to be organized into nucleosomes containing acetylated histones, which results in a more accessible, open chromatin. Conversely, methylated DNA sequences are correlated with nucleosomes that include non-acetylated histone H3 and H4, thereby creating a more compact and less accessible chromatin structure [28–30]. Recent study reveals an impact of H3K4me3 on DNA methylation patterning, highlighting a critical DNMT ATRX-DNMT3-DNMT3L domain-H3K4 interaction in humans. Their model also indicates DNA methylation is generally suppressed by H3K4me1/3 and H3K27me3, but increased in H3K9me3 and H3K36me3 regions [31]. Hypoxia can influence epigenetic enzymes in several ways. Many enzymes such as ten-eleven translocation (TET) enzymes, which is involved in DNA demethylation [32], and JmjC domain-containing histone demethylases, which is involved in histone demethylation [33] are oxygen-dependent dioxygenases. Under hypoxic conditions, the activity of these enzymes is reduced and potentially leading to hypermethylation of specific DNA regions and altered histone methylation patterns.

### Non-coding RNA (ncRNA) and micro RNAs

Non-coding RNAs (ncRNAs) are functional RNA molecules that are transcribed from DNA but are not translated into proteins [34]. NcRNAs include microRNAs (miRNAs), long ncRNAs (lncRNAs), and circular RNAs. They regulate gene expression via post- translational and epigenetic mechanisms. miRNAs are a diverse group of small, non-coding RNAs, approximately 22 nucleotides long, that regulate gene expression by binding to specific target sites on mRNAs.

Modulating microRNA (miRNA) activity using miRNA analogs, miRNA-based therapeutics, or anti-miRNAs is a compelling and actively pursued strategy for cancer treatment. The clinical viability of nucleic acid-based therapies is further underscored by the FDA approval of patisiran, a small interfering RNA (siRNA) designed to target and degrade transthyretin mRNA for the management of rare polyneuropathy [35]. LncRNAs, which are > 200 nucleotides in length, do not encode proteins but play crucial roles in regulating gene expression. They can regulate gene expression via cis-/trans-interactions with chromatin factors, RNA-binding proteins, and enhancers, as well as by modulating miRNA functions. Our group previously showed that the LncRNA microRNA-210 host gene (*MIR210HG*) exhibits rapid upregulation following hypoxic conditions, and it facilitates hypoxia-inducible factor 1 (HIF1) α expression through competitive binding and subsequent downregulation of micro RNA-93-5p. *MIR210HG* is likely to play a significant role in the cellular response to hypoxia within renal tubular epithelial cells [36].

## Epigenetic changes caused by AKI

AKI can be induced by multiple factors, including ischemia–reperfusion injury (IRI), acute loss of renal function, sepsis, and nephrotoxic chemotherapy (e.g., cisplatin and immune checkpoint inhibitors) [37]. Renal injury leads to cell death and inflammation. The body attempts to repair the damage through cell dedifferentiation, proliferation, and redifferentiation. Although this process can sometimes lead to the full recovery of kidney function and structure, it can also result in CKD and end-stage renal disease (ESRD) [38]. Tubular epithelial cells (TECs) in the kidney are considered the primary target of AKI because of their susceptibility to various types of stress, such as ischemia-induced hypoxia and nephrotoxic substances [39]. Hypoxia plays an important role in the pathogenesis of kidney disease. The unique physiology of the kidney, characterized by a diffusional oxygen shunt between its arterial and venous vessels, results in a relatively low oxygen supply. This inherent susceptibility to oxygen deprivation makes the kidneys particularly vulnerable to hypoxic stress, which exacerbates kidney injury and disease progression [40, 41]. Histone acetylation is a key epigenetic modification involved in AKI and subsequent repair processes, and it has been functionally linked to DNA replication, repair, and gene transcription [42]. In this part, we focus on histone modifications in AKI and the AKI-to-CKD transition. Substantial evidence suggests that AKI is associated with alterations in histone acetylation, particularly in the context of ischemic AKI.

### Histone deacetylase and histone acetyltransferase play crucial roles in AKI

Histone acetylation is altered in AKI and is induced by various factors. Kidneys damaged by ischemia have the potential to regenerate via a mechanism involving intrarenal induction of protective factors, including bone morphogenetic protein-7 (BMP7). During recovery after transient energy depletion in epithelial cells, HDAC5 is downregulated, leading to increased histone acetylation and the subsequent upregulation of the *BMP7* gene. This coordinated response has been observed in both cell cultures and animal models of kidney ischemia. These findings suggest that targeting HDAC5 could potentially enhance kidney repair and regeneration by promoting the expression of protective factors, such as BMP7 [43].

In healthy kidneys, the jade family PHD finger 1 (JADE1) protein functions as a key regulator of cellular oxygen-sensing pathways. JADE1 and histone acetyltransferase HBO1 were found in the nuclei of tubular cells. After kidney injury, the levels of these proteins initially decrease but recover during kidney repair. JADE1 mRNA produces two protein products: full-length JADE1L consisting of 842 amino acids and its truncated splice variant, JADE1S, which lacks a large C-terminal fragment of 333 amino acids. JADE1S specifically associates with acetylated histone H4, suggesting a role in regulating gene expression during cell proliferation. These findings indicated that the JADE1-HBO1 complex plays a crucial role in kidney recovery after injury, with different isoforms potentially having distinct functions [42].

In addition, modifications to histones at the HMG-CoA reductase (*HMGCR*) gene promoter contribute to increased HMGCR expression. The upregulation of HMGCR may have a protective effect against ischemic acute kidney injury [16]. Furthermore, activating transcription factor 3 (ATF3) binds to HDAC1 and recruits it to ATF/NF-κB sites in the IL-6 and IL-12b gene promoters. This interaction leads to histone deacetylation, which condenses chromatin structure, preventing the binding of the transcription factor NF-κB and inhibiting the expression of inflammatory genes following ischemia–reperfusion injury [44].

Conversely, studies have demonstrated that increased global H3K9/18 acetylation, particularly in inflammatory genes such as *Ccl2*, *Icam-1*, and *Vcam-1*, and locus-specific H3/4 acetylation in genes such as *Il6, Il12b,* and *Serpine1*, is associated with upregulated gene expression in mouse models of ischemia–reperfusion (IR) and lipopolysaccharide (LPS)-induced AKI. These findings suggest a regulatory role of histone acetylation in inflammation-related processes during AKI [44–46].

### Histone demethylase and histone methyltransferase play roles for AKI and kidney repair

Histone methylation plays a crucial role in regulating gene expression by influencing chromatin structure and function. Unlike histone acetylation, the role of histone methylation in AKI and kidney repair remains relatively unknown. The methylation of histone lysine or arginine residues is modulated by enzymes known as methyltransferases and demethylases. Methyltransferases are categorized into two main types: lysine methyltransferases (KMT) and arginine methyltransferases (RMT) [45]. A histone methyltransferase responsible for trimethylating lysine 27 on histone H3 (H3K27m3) has been identified in the fibrotic kidneys of both unilateral ureteric obstruction (UUO) mice and patients with CKD. This suggests that methyltransferases have profibrotic functions [47, 48]. In addition, histone 3 lysine 9 acetylation (H3K9Ac) and trimethylation (H3K9Me3) increased after UUO in mice. The increase of H3K4me3 methylation following IRI was strongly linked to the increased expression of genes involved in inflammation (*TNF-α, CCL2*), fibrosis (*TGF-β1, type III collagen*), and cholesterol regulation (*HMGRC*), ultimately contributing to the development of CKD [49, 50]. On the other hand, it is studied that histone 3 lysine 4 dimethylation (H3K4Me2) increased in diabetic nephropathy in uninephrectomy db/db mice, and decreased in uninephrectomy C57BL/6 mice, suggests that advanced diabetic nephropathy is associated with increased renal H3K9 and H3K23 acetylation, H3K4 dimethylation and H3 phosphorylation at serine 10 that enhance chromatin unfolding and gene expression [51].

Not only histone demethylase, methyltransferases also contributes to increased kidney fibrosis. Enhancer of zeste homolog 2 (EZH2), a methyltransferase that induces histone H3 lysine 27 trimethylation (H3K27me3), has been reported to be increased in the fibrotic kidneys of mice with UUO and in patients with CKD, suggesting that this methyltransferase has profibrotic functions. In AKI models induced by IR and folic acid (FA), inhibition of EZH2 with 3-deazaneplanocin A (3-DZNeP) protects renal tubular cells from injury and death. The development of renal fibrosis is accompanied by increased expression of EZH2 and vimentin, mesenchymal markers, and decreased expression of E-cadherin, phosphatase and tensin homolog (PTEN), and Smad7. Pharmacological inhibition of EZH2 stabilizes the expression of E-cadherin, PTEN, and Smad7 and prevents the upregulation of vimentin in the kidney after UUO injury. In summary, these findings highlight EZH2 as a potential therapeutic target for acute and chronic kidney diseases [47].

### Roles of other histone modifications in AKI

Recent mass spectrometry-based proteomic studies have uncovered a wide range of novel histone lysine acylations, including propionylation, butyrylation, 2-hydroxyisobutyrylation, β-hydroxybutyrylation, malonylation, succinylation, crotonylation, glutarylation, and lactylation [52].

Histone crotonylation is a recently described post-translational modification. This process involves the addition of a crotonyl group to lysine residues on core histones via the action of histone crotonyltransferases. Similar to histone acetylation, histone crotonylation neutralizes the positive charge on lysine residues and stimulates transcription [53]. It is reported that histone crotonylation might modulate kidney injury. Histone crotonylation has been studied in cultured murine proximal tubular cells and the kidneys of mice with FA-or cisplatin [54].

## Epigenetic changes caused by the pathophysiological finding in AKI-to-CKD transition

Recent studies have suggested a strong association between AKI and CKD development. Several clinical studies have shown that some individuals, particularly those with severe or recurrent AKI, have a significantly increased risk of developing CKD [55]. AKI can lead to various structural changes in the kidney, including nephron loss, incomplete tubular repair, vascular rarefaction, interstitial inflammation, and shifts in the interstitial cellular composition. Collectively, these changes contribute to CKD progression [56]. AKI-to-CKD transition is mediated by the interplay between tubular epithelial cells, endothelial cells, pericytes, inflammatory cells, and myofibroblasts. Renal hypoxia exacerbates these processes and plays a significant role in disease progression [9, 57, 58].

Alterations in CKD lead to a state of relative hypoxia, even under basal conditions, which is characterized by a diminished number of peritubular capillaries [59, 60]. Microvascular loss occurs along with increased fibrosis, leading to increased relative hypoxia within the kidney and in particular within the outer medulla [61]. Kidneys afflicted with CKD demonstrate increased RAS activation. A diminished number of glomeruli results in hyperfiltration and increased tubular oxygen consumption in the corresponding tubules, further exacerbating the imbalance between oxygen requirement and delivery [62].

The peritubular capillaries surrounding the renal tubules have a unique structure that allows oxygen to bypass parallel arterial and venous vessels. This reduces the amount of oxygen that the kidneys can extract, rendering them more susceptible to oxygen deprivation [40]. Because of the limited capacity of tubular cell proliferation, proximal tubular cells are thereforevulnerable to ischemic injury, which is the leading cause of AKI, and are considered the primary target of AKI, and increased severity of AKI leads to maladaptive tubular repair, which predisposes to fibrosis and CKD [39, 63]. The renal microvasculature, unlike the renal tubules, have limited ability to repair themselves. This makes them vulnerable to damage from AKI, leading to a reduction in the number of capillaries, a condition known as “capillary rarefaction.” This reduction in the number of blood vessels results in chronic renal hypoxia, which contributes to CKD progression [56, 64]. Several mechanisms may underlie this capillary rarefaction, including decreased production of the vascular endothelial growth factor by tubular epithelial cells and detachment of pericytes localized close to the endothelial cells that maintain vascular stability [57, 58].

During the course of AKI-to-CKD transition, hypoxia-induced epigenetic changes are recorded in the cell that have a long-term effect, known as “hypoxic memory” [40, 65]. Hypoxia also stimulates inflammatory cells, including immune cells like macrophages and neutrophils. These cells play roles in tissue repair and scar formation, leading to renal fibrosis [66, 67].

Hypoxic stimulation also changes the chromatin conformation via the complex of HIF1 and histone demethylase. Our group discovered that hypoxia-inducible factor 1 (HIF-1) and lysine-specific demethylase 3 A (KDM3A) work together to regulate the expression of the solute carrier family 2A3 (SLC2A3) gene through changes in chromosome conformation [68, 69]. Under normal oxygen conditions, a specific region upstream of the SLC2A3 promoter forms a loop with the help of HIF-1. This loop disappears when HIF-1 is inhibited. However, under hypoxia, KDM3A is recruited to the SLC2A3 locus, and changes in the chromosomal conformation lead to increased gene expression [68, 70]. Additionally, we exposed cultured tubular cells to hypoxia and identified genes that are directly regulated by HIF-1 through a genome-wide analysis of HIF-1 binding sites. We identified 44 long non-coding RNAs (lncRNAs) whose expression was upregulated under hypoxia in two different types of tubular cell lines. One of these lncRNAs, DARS-AS1, is specifically upregulated under hypoxia and HIF-1 binds to its promoter region. Functionally, DARS-AS1 protected renal tubular cells from apoptosis [71]. These results show that hypoxic stimulation may induce persistent gene expression via chromatin conformational changes or long non-coding RNAs.

## Update on the epigenetics of histone modifications in hypoxic memory

With respect to hypoxic memory, a number of hypoxia-induced epigenetic changes have been reported in numerous studies and widely implicated in gene/histone modification under hypoxic conditions (Table 1).

**Table 1:** Epigenetics in hypoxic memory

Model	Histone post-translational modifications	Modified gene	Observations/Findings	Contribution to AKI-to-CKD Transition	PMID
IRI mouse model	Increased H3K4me3, H2A.Z	*Ccl2, Tgfb1, Col3a1*	Increased levels of H3K4me3 and/or H2A.Z at profibrotic genes (Tgfb1, Col3al) sustained for 7 d. BRG1 is essential for transcriptional activation of TNF-a and MCP-1	Epigenetic modification driven by Set1 and BRG1 activation contributes to sustained overexpression of profibrotic genes	19261745 [71]
Glycerol-induced AKI mouse model	Increased H3K4me3, H2A.Z	*Tnf, Ccl2, Tgfb1*	Progressive increase in H2AZ levels observed at 1, 2, and 7 d after glycerol injection	Sustained increase in H2AZ levels contributes to the progression of AKI-to-CKD	20032114 [74]
IRI mouse model	Increased H3K4me3, H2A.Z	*Tnf, Ccl2*	The expression of *TNF-α* and *MCP-1* genes significantly increased and persisted for a 1w after AKI followed by IRI	BRG1 is essential for transcriptional activation of *TNF-a* and *MCP-1*	19556365 [75]
IRI mouse model	Increased H3K9/14ac	*Lcn2, Ccl2, Tnf, Tgfb1*, *Col3a1, Hmgcr*	Progressive decline in kidney function over three weeks. Increased pro-inflammatory cytokines/chemokines (MCP-1, TNF-a, TGF-β1) and decreased anti-inflammatory factors were observed	Sustained activation of inflammatory and fibrotic pathways driven by increased histone acetylation of proinflammatory and profibrotic genes contributes to progression of AKI-to-CKD	21921025 [76]
Nephrotoxic AA and FA mouse models	Decreased H3 acetylation	*GPX4/Gpx4*	Elevated HDAC3 levels and decreased *GPX4* expression caused renal tubular epithelial ferroptosis from 3 to 14 d post-treatment. HDAC3 and KLF5 interact to suppress *GPX4* by binding to its promoter and inducing histone deacetylation	Epigenetic mechanism involving HDAC3 and GPX4 drives ferroptosis and renal damage in AKI-to-CKD	37890360 [77]
ANCA-associated glomerulonephritis patients, mouse models	Increased H3K27me3	*PTEN*	Increased EZH2 expression and H3K27me3 levels persisted for 4 w after AKI. EZH2 binds to PTEN promoter and suppresses its expression, activating downstream pathways (EGFR, ERKl/2, STAT3)	EZH2 plays a critical role in the AKI-to-CKD transition by regulating multiple cellular processes, including EMT, cell cycle arrest, and inflammation	37029114 [78]
IRI Mouse model	Decreased H3K27me3	*Col3a1*, *Col4a1*, *TIMP2*, *MMP14*	DZNep (H3K27me3 inhibitor) reduced tubulointerstitial fibrosis persistent 8 w after AKI. DZNep decreased pro-fibrotic genes (COL3A1, TIMP2, COL4A1, MMP14)	Pharmacological modulation of H3K27me3 may be a promising therapeutic strategy for renal fibrosis	29491489 [79] 37710084 [80]
IRI mouse model	differentially methylated CpGs	Hypermethylation at *2700049A03Rik, Ccr9*	Differential methylation patterns observed in promoter regions persisted at 24 h and 7 d	Persistent promoter methylation after IRI may contribute to AKI-to-CKD transition	28189760 [81]
IRI + right nephrectomy rat model	Hyper-methylation	HIFlα binding sites at *Vegfα* promoter	Significant increase in oxidative stress and decrease in global DNA methylation observed after IRI. HIFla initially suppressed, recovered later. VEGF expression remained low. Hypermethylation of VEGF promoter at HIFlα binding site observed early	Early events like oxidative stress, DNA methylation changes, and impaired HIF1a/VEGF signaling play a crucial role. Targeting these early mechanisms may prevent CKD progression	33888767 [84]

AA, antimycin A; AKI, acute kidney injury; ANCA, anti-neutrophil cytoplasmic antibody; BRG, also known as BRM; CKD, chronic kidney disease; Col3a1, collagen type III alpha 1 chain; Col4a1, collagen type IV alpha 1 chain; EGFR, epidermal growth factor receptor; ERKl/2, extracellular signal-regulated kinase 1/2; FA, folic acid; Gpx4, glutathione peroxidase 4; HDAC3, histone deacetylase 3; HIF1, hypoxia-inducible factor 1; Hmgcr, 3-hydroxy-3-methylglutaryl-CoA reductase; IRI, ischemia reperfusion injury; KLF5, Krüppel-like factor 5; MMP14, metalloproteinase 14; PTEN, phosphatase and tensin homolog; PMID, PubMed Identifier; SET1, SET domain containing 1; STAT3, Signal Transducer and Activator of Transcription 3; Tgfb1, transforming growth factor β 1; TIMP2, tissue inhibitor of metalloproteinase 2; VEGF, Vascular Endothelial Growth Factor

Our previous study showed that renal hypoxia contributes to the pathophysiology of the AKI-to-CKD transition [72]. In mouse models with IRI, increased levels of H3K4me3 and/or histone 2 variant H2A.Z were observed at the profibrotic genes transforming growth factor-β1 (*Tgfb1*) and collagen III a1 (*Col3a1*). This epigenetic modification is driven by the activation of two relevant histone-modifying enzymes, H3K4 methyltransferase Set1 and the chromatin remodeling enzyme BRG1. Overexpression of these genes was sustained for 7 days post-IRI [73]. Also, in a glycerol-induced AKI mouse model, a progressive increase in H2AZ levels was observed at 1, 2, and 7 days after glycerol injection. In addition, increase of TNF-α, monocyte chemoattractant protein-1 (MCP-1), and Tgfb1 mRNAs, Pol II gene binding were continuously observed after 7 days recovery from AKI [74]. In other study, the expression of *TNF-α* and *MCP-1* genes significantly increased and persisted for a week after AKI followed by IRI. Increased levels of RNA Pol II in these genes indicated that the elevated mRNA levels were due to increased transcription. BRG1 binding to the *TNF-α* and *MCP-1* genes closely mirrored Pol II recruitment. Knockdown of BRG1 in tubular cells inhibited the upregulation of TNF-α and MCP-1 in response to ATP depletion, suggesting that BRG1 is essential for their transcriptional activation. Overall, the study demonstrates that BRG1 plays a crucial role in promoting the transcription of *TNF-α* and *MCP-1* genes in kidney tubular cells following IRI [75]. In another study on AKI-to-CKD in a 30-min IRI model, mice exhibited a progressive decline in kidney function over three weeks. This was characterized by increased expression of pro-inflammatory cytokines and chemokines (MCP-1, TNF-α, TGF-β1) and decreased expression of anti-inflammatory factors (heme oxygenase-1, IL-10). Additionally, increased collagen III mRNA levels and collagen deposition indicate a progressive profibrotic response. The authors suggested that these changes were driven by epigenetic modifications, specifically the increased histone acetylation of proinflammatory and profibrotic genes. This sustained activation of the inflammatory and fibrotic pathways contributes to the progression of AKI to CKD [76]. Further study investigates the role of histone deacetylase 3 (HDAC3) -mediated ferroptosis in the progression of AKI to CKD. Elevated HDAC3 levels and decreased glutathione peroxidase 4 (GPX4) expression caused renal tubular epithelial ferroptosis, which was observed in mouse models induced by nephrotoxic aristolochic acid (AA) and folic acid (FA), from 3 d post-treatment in the AKI phase to 14 d post-injection CKD phase. HDAC3 inhibition reduces ferroptosis and renal fibrosis. HDAC3 and Kruppel-like factor 5 (KLF5) transcription factors interact to suppress GPX4 expression by binding to its promoter and inducing histone deacetylation. This study highlights a novel epigenetic mechanism involving HDAC3 and GPX4 that drives ferroptosis and renal damage in AKI-to-CKD [77].

In another study on AKI-to-CKD transition, histone methyltransferase was found to be involved. Increased enhancer of zeste homolog 2 (EZH2) expression and H3K27me3 levels were observed in kidney tissues of patients with ANCA-associated glomerulonephritis and in mouse models, which persisted for 4 weeks after AKI. EZH2 binds to the promoter of the phosphatase and tensin homolog *(PTEN)*, a tumor suppressor gene, and suppresses its expression. This leads to the activation of downstream signaling pathways, including the epidermal growth factor receptor (EGFR), extracellular signal-regulated kinase 1 and 2 (ERK1/2), and signal transducer and activator of transcription 3 (STAT3), which promote epithelial-mesenchymal transition (EMT), cell cycle arrest, and inflammation. EZH2-mediated EMT results in the loss of renal tubular epithelial cell transporters and promotes M2 macrophage polarization, further exacerbating kidney injury. This study suggests that EZH2 plays a critical role in the AKI-to-CKD transition by regulating multiple cellular processes, including EMT, cell cycle arrest, and inflammation [78]. In addition, about the study investigated the potential of targeting epigenetic modifications to treat renal fibrosis, The drug DZNep, which inhibits the H3K27me3 histone modification, was found to reduce tubulointerstitial fibrosis in a mouse model of AKI-to-CKD transition, persistent 8 weeks after AKI induced by IRI. DZNep treatment decreased the expression of pro-fibrotic genes such as *COL3A1*, *TIMP2*, *COL4A1*, and *MMP14* in both in vivo and in vitro experiments. This study suggests that DZNep exerts its antifibrotic effects by altering the epigenetic landscape of kidney tubular cells. These findings indicate that pharmacological modulation of epigenetic modifications, specifically targeting H3K27me3, may represent a promising therapeutic strategy for the treatment of renal fibrosis [79, 80].

## Update on the epigenetics of DNA methylation and other modifications in hypoxic memory

In addition to histone modifications, changes in DNA methylation can persist for several days. A previous study investigated the role of DNA methylation in IRI pathophysiology. Reduced-representation bisulfite sequencing revealed a significant decrease in genome-wide DNA methylation levels, including those in the promoter, CpG island, exon, and intron regions, in renal tissues after IRI. In addition, hundreds of genes showed differential methylation patterns in the promoter regions at different time points after IRI, which persisted at 24 h and 7 days post-IRI. A subset of these genes, including 2700049A03 Rik, chemokine (C–C motif) receptor 9 *(Ccr9*), familial glucocorticoid deficiency type 2 (*Fgd2*), 6-phosphofructo-2-kinase/fructose-2,6-biphosphatase 3 (*Pfkfb3*), and syndecan 4 (*Sdc4*), exhibited altered promoter methylation and corresponding changes in gene expression in an independent cohort of renal tissues [81].

It can be assumed that persistent promoter methylation in IRI contributes to epigenetic memory during the AKI-to-CKD transition. A study performed parallel single nucleus RNA-sequencing (snRNA-seq) and single nucleus assay for transposase-accessible chromatin using sequencing on adult human kidneys, and they characterized the transcriptional activity and chromatin accessibility. Their findings show that both methods are comparable for identifying cell types, and that cell-type-specific chromatin accessibility offers valuable extra details, improving the understanding of cellular diversity [82]. In addition, recent research by single nucleus RNA sequence reveals that even moderate IRI is linked to persistent injury spreading from the cortico-medullary boundary to the cortex, and remaining failed-repair PTCs are probable triggers for CKD [83]. These studies demonstrate that scRNA-seq is a very useful tool to reveal cell type-specific gene expression changes and the role of specific cell populations in the AKI-to-CKD transition. Since the epigenomics of each cell type is different, the bulk analysis does not distinguish between various cell types, which makes it difficult to understand cell-specific epigenetic changes. For example, Bulk DNA methylation demand considerable amounts of DNA, typically sourced from diverse cell populations, resulting in an aggregated methylation level rather than accurate data for rare, individual cells [84]. However, with the development of the single cell technique, such as single-cell DNA methylome sequencing, single-cell ChIP-seq, and single-cell Hi-C, it is expected to be possible to compare the differences between normal conditions and diseased state within the same cell type. They have recently been significantly improved in the areas of cancer and embryonic development. For example, recent study used single-cell Hi-C to map chromosomal conformations in post-gastrulation mouse embryo cells, to understand the balance between cell proliferation and cell fate determination during embryonic development [85]. In addition, there is a study which demonstrates that single-cell ChIP-seq method is useful for analyzing chromatin state heterogeneity at single-cell resolution within complex biological systems such as tumors [86]. Therefore, single cell techniques and transcriptome analysis can be the solution for cellular heterogeneity.

Moreover, there was a significant increase in oxidative stress and a decrease in global DNA methylation after IRI. The expression of HIF1α and VEGF, key players in the hypoxic response, was initially suppressed. While HIF1α expression recovered later, VEGF expression remained low. Hypermethylation of the VEGF promoter gene at the HIF1α binding site was observed early on, suggesting a potential mechanism for the persistent suppression of VEGF expression. These findings suggest that early events, such as oxidative stress, DNA methylation changes, and impaired HIF1α/VEGF signaling, play a crucial role in the transition from AKI to CKD. Targeting these early mechanisms may offer therapeutic opportunities for preventing CKD progression [87]. As a recent topic, the clustered regularly interspaced short palindromic repeats (CRISPR)-Cas9 system, originally identified as an adaptive immune system in bacteria, has emerged as a revolutionary gene-editing tool. Its programmability, using a guide RNA (gRNA) to target specific DNA sequences, has been transformative. The CRISPR/Cas9 system can edit the epigenome, such as adding tags or notes to the DNA that tell genes when to turn on or off, without changing the DNA letters themselves [88]. Significant progress is noted in the development of a diverse toolkit of epigenome editors (writers, erasers, activators, repressors) and their successful application in functional genomics, disease modeling, and initial therapeutic explorations [89]. The potential to treat a broad range of human diseases, particularly those with epigenetic issues or where gene expression modulation is beneficial, is highlighted.

## Limitations

It is unknown that whether epigenetic changes are drivers or consequences of cellular phenotypes. Specifically, epigenetic changes are induced by hypoxia as a consequence. Those epigenetic changes as drivers can trigger another epigenetic change.

## Conclusion

**Fig. 1:**
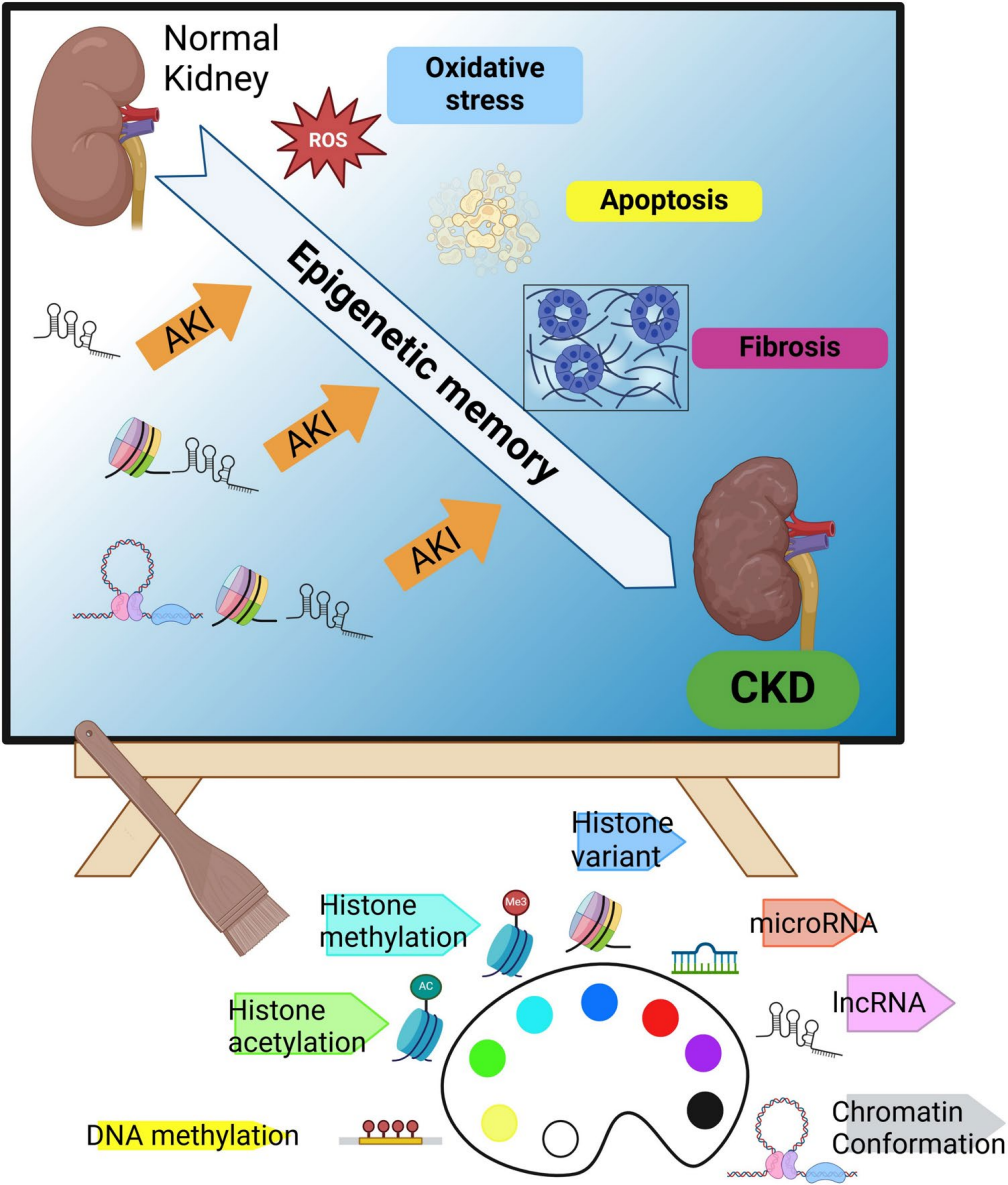
Repeated AKIs lead to CKD, modified by epigenetic marks. During the AKI-to-CKD transition, a subset of AKI-induced epigenetic alterations persists in cells, potentially driving the progression of tubulointerstitial fibrosis. “Epigenetic memory” refers to the stable retention and transmission of unique gene expression patterns to daughter cells, encoded by epigenetic marks and associated epigenetic memory factors. The accumulation of these epigenetic memories contributes to irreversible CKD. This figure schematically depicts the concept of hypoxic memory, focusing on the pathophysiology of the AKI-to-CKD transition, using the analogy of a canvas and a palette of paints. In this representation, different colors in the palette symbolize various epigenetic modifications, such as histone modifications, DNA methylation, and microRNAs. Just as an artist blends colors with a brush to create a painting, multiple epigenetic modifications interact to shape disease progression. The canvas illustrates the gradual progression to CKD as recurrent AKI episodes occur. During the AKI-to-CKD transition, repeated AKI events lead to irreversible conditions such as fibrosis through oxidative stress, inflammation, and apoptosis

In the AKI-to-CKD transition, depending on the severity of AKI, a subset of AKI-induced epigenetic alterations remains in the cells, which may be crucial drivers mediating the subsequent progression of tubulointerstitial fibrosis. It is also evident that changes in DNA methylation and histone modifications within kidney cells and their surrounding environment play a significant role in the progression of kidney disease. (Fig. 1) Hypoxic memory can transform transient environmental factors or events into long-term drivers of pathogenic changes. Targeting epigenetic mechanisms could be a promising therapy for the AKI-to-CKD transition. Identifying the detailed mechanisms of epigenetic memory and considering temporal specificity are crucial for developing effective treatments.
